# Development of Antimetastatic Drugs by Targeting Tumor Sialic Acids

**DOI:** 10.3797/scipharm.1205-01

**Published:** 2012-06-18

**Authors:** Da-Yong Lu, Ting-Ren Lu, Hong-Ying Wu

**Affiliations:** 1 School of Life Sciences, Shanghai University, Shanghai 200444, China.; 2 College of Science, Shanghai University, Shanghai 200444, China.

**Keywords:** Sialic acid, Neuraminidic acids, Neoplasm metastases, Anticancer therapy, Neoplasm targeting, Probimane, Sialylation, Glycosylation, Cancer chemotherapy, Glycobiology, Cancer biology

## Abstract

One-third of all cancer categories in clinics have a high incidence of neoplasm metastasis. Neoplasm metastasis is one of the leading causes of cancer deaths. However, the prevailing therapeutic approach to this pathogenic process is presently unsatisfactory. Paradoxically to our efforts and expectations, except for some antibodies, no obvious improvements and therapeutic benefits in currently used drugs have been achieved until now. Therapeutic benefits in late-stage or elderly cancer patients are especially poor and useless. One of the reasons for this, we would guess, is the lack of therapeutic targets specifically related to neoplasm metastasis. In order to enhance the therapeutic efficacy, the development of antimetastatic drugs transcending from current drug-screening pathways is urgently needed. Antimetastatic drugs targeting aberrantly sialylated in tumors have evolved for about a quarter of a century and might be a future therapeutic option other than the currently utilized antimetastatic drugs, such as antivascular and MMP inhibitors. Since neoplasm tissues often manifest high levels of sialic acids and sialyl antigens or glycoligands, some types of sialic acid analogue, such as N-glycolylneuraminic acid (Nau5Gc), occurred in most tumor tissues which is normally absent in most humans. Consequently, more attention is needed to work with new therapeutic approaches to target these changes. This review addresses and discusses the latest six types of therapeutic approaches targeting sialic acids in metastatic tissues.

## Introduction

Cancer has one of the highest-mortality rates, which places it in the top five causes of annual deaths in almost all countries. Unlike cardiovascular diseases, the treatment beneficiary for cancers—especially for epithelial carcinoma—has been improving slightly over the past several decades [1–3]. Neoplasm metastasis is one of the most fatal characteristics responsible for these unsatisfactory anticancer therapies—more than 60% of cancer deaths—and can only be hopefully controlled by drugs. Paradoxically to our efforts and expectations, except for some antibodies, no obvious improvements and therapeutic benefits in conventional antimetastatic drugs (usually antivascular agents or MMPs inhibitors) have been achieved until now. Therapeutic benefits in late-stage or elderlycancer patients are especially poor and useless [1–3]. Clinical anticancer drug therapies currently in use have been mainly focusing on primary tumor growth, rather than specifically targeting the pathologic courses of metastases. Finding important drugs targeting specific neoplasm metastases is essential and indispensable [4–6]. It nevertheless requires changing our focus from targeting tumor vascularity and MMPs [4] into more metastatic-relating molecules [5, 6].

According to a general point of view, good antimetastatic therapy must be based on thorough understanding of metastatic biology and pathology. Antimetastatic drugs extensively studied nowadays have been mainly focusing on angiogenesis and MMPs inhibitors [4]. However, these two types of agents are far from satisfactory and generally offer only a few months of survival benefits. In order to make a definitive breakthrough from this stalemate, novel ideas and even some shotgun-like molecular expeditions of a drug that is therapeutically related with metastasis itself, seem to be a future trend [6]. One of these novel targets has been aberrant sialylation in neoplasm tissues [7–10]. It is not a well-studied therapeutic target. In this review, important targets at sialylation alterations in neoplasm tissues have been documented, described, discussed, and highlighted, and drug developments based on the above-mentioned pathways are suggested.

## Relationship between Cancer Malignancy and Sialic Acids Levels

Sialic acids (Sias, neuraminic acid) are a special series of 9-carbon backbone acidic carbohydrates and are typically found at the outermost part of sugar chains attached to cell membrane macromolecules. They play many important roles in a series of physiological and pathologic processes, including microbe binding that leads to infections, regulation of the immune response, the progression and spread of human malignancies, and in certain aspects of human evolution [11–13]. The earliest work tackling the phenomenon of a positive relationship between sias and tumors can be traced back to Kimura et al from 1958 [14, 15]. Their discovery proposed that tumor cells might excrete and contain more sialyl glycans, glycoproteins, or glycolipids. These characteristics were later found to link with highly metastatic tumor types [16]. It has been shown that there is higher total sias content in highly metastatic tumor cell lines, than those in lower metastatic tumor cell lines. Since then, numerous similar reports and reviews have rapidly been published. Novel ideas have been proposed and can be mainly concluded as follows:

## Mechanisms and Different Pathways of Anticancer Drugs on Tumor Sialic Acids

Many research studies have shown patients with tumors that have high levels of sialyl Lewis X antigens, which appear to be linked with a poor prognosis for patients, which is one of the most conspicuous pathologic features of sias in tumors clinically [17, 18]. Similarly, the sialyl Tn antigen has been found to be higher in metastatic cancer tissues, and people carrying these sialyl Tn antigen cancer tissues tend to have poor outcomes during pregnancy [19, 20]. Many relationships between neoplasm metastasis and sias aberration can be seen from reviews [7–10]. These pathways should be regarded as important targets for drug therapies.

Since the antimetastatic therapy targeting neoplasm sias is greatly under-investigated, we can only provide a few citations based on different mechanisms of action. By investigating these citations, we extract the following six pathways for future research.

More than 50 different types of sias monosaccharides have ever been discovered, which can be linked with other normal monosaccharides (heptoses or hexoses and so on) to form tremendously diversified 2–6 sugar component antigens (sugar chains)—sias are often at the farthest end of antigens and glycoproteins. Since sias consists of more than 50 sias sugars (monosacchrides) [21], are there similarities and differences in the biological activities between these sias-analogues? On the other hand, a different linkage of sias with other sugars, e.g. a N- or different O-linkage, will show different biological or pathogenic actions. The different sias derivatives, linkages, conjugates, and poly-saccharides from pathophysiological sias in tumors might be antagonists for tumor growth or even metastases [22–26]. Owing to the same type of sugars (sialic acids) in structure in the body, they will competitively bind to remote normal tissues and organs, which ought to bind to tumor cells and thus fulfill neoplasm metastases. Or these sias-conjugates will compete with sias for key enzymes in neoplasm tissues and make these enzymes less effective or nullified. They will therefore inhibit neoplasm metastasis. For example, a sias-conjugate has been reported to inhibit pulmonary metastases of a mouse colon adenocarcinoma [22, 23]. These data have been done *in vitro* or in mice, and there have been no systematic clinical data. We must emphasize and encourage more of these studies in clinics. These agents have been found to be almost of no toxicity to humans, so only low hepatotoxicity of sialyl polysaccharide after long-term application has been found in a 1^st^ phase clinical study [25]. A disaccharide precursor of sialyl Lewis X, though, links with the biology of cancer malignancy, and can inhibit tumor metastasis *in vitro* [26]. It implies that the sialyl Lewis X antigen can have feedback activity in the cell carcinogenesis or metastasis.

Since many sias-conjugates or sialyl antigens can inhibit tumor metastasis, sias-conjugates can be regarded as potential therapeutic agents for treatments of neoplasm metastasis. We can synthesize a series of similar compounds to be tested as anticancer or antimetastatic drugs and further study their anticancer and antimetastatic mechanisms. There must be novel therapeutic targets waiting to be clarified.

For all the reasons we state above, we must pay more attention to the regulation and functions of sias analogues in cells, especially in cancer cells. All of the differences between linkages and substitutions of sias can be recorded by a glycome [27–31]. Since more than 50 types of sias have been discovered, with some of them oncologically related, the biologically important sias analogues or -conjugates should be future therapeutic targets.

To evaluate the possibility of sias in tumors as an anticancer or antimetastatic target, we have systematically carried out 13 first-line or second-line anticancer drug pharmacological evaluations in mice for building the relation between sias inhibitions and the treatment outcomes of drugs. We used a colorimetry method to detect sias contents in the sera of mice bearing tumors. Our experiment was to study if anticancer (especially antimetastatic) drugs can inhibit sias levels in mice bearing tumors [32, 33]. Our work shows that some of the anticancer drugs can more significantly inhibit serum sias levels in mice bearing the tumors S180 and Lewis lung carcinoma. From these four antineoplastic drugs, probimane is an antimetastatic agent and a DNA-chelating agent [6]. Cisplatin and nitrogen mustard are well-known DNA chelating agents, and lycobetaine is also a DNA-binding agent [34]. So, it is proposed that inhibitions of tumor sialic acids by these drugs might be through a DNA template via two ways.

1. DNA→RNA→proteins (CMP-sialic acid synthase, sialyltransferases and sialidase)
2. DNA→unknown mechanisms→sialyl-conjugators

It might be through an unknown mechanism to fulfill this mechanism and needs our further work.

However, many cytotoxic agents that target tumor metabolism showed no inhibition of serum sias levels in mice bearing tumors. Similar to our standpoint, Abde-Hamid NM et al reported that some anticancer drugs that did not show typical antimetastatic effects, such as 5-Fu, could not inhibit sia levels in tumor cells [35]. These works show that the inhibitions of sias in tumors can be a good model to study antimetastatic drugs, rather than antiproliferative drugs, and can be of significance for studying their underlying mechanisms. This is a relatively new therapeutic target for us to study.

To consider the possible routes for tumor cells to accumulate sias, one might immediately relate them with enzymes. Human sialyltransferases and sialidases as cancer markers and drugs’ targets have also been suggested. All of the differences between linkage and substitutions of sias might be the results or aftermath of the activity and contents of sialyltransferase or sialidase changes in tumors. It adds to the complexity, volume, and intensity of research. Future new technologies for specific detections of these enzymes and their activities will help us to better understand these relations. Now, many sialyltransferases or sialidases have been found to be expressed relatively higher or lower in tumors than in normal tissues [36–38]. The differences in sialyltransferase and sialidase manifestations between these normal cells and cancer cells can help us to better understand sias biology and pathology in cancer. This is an important route in order to explore agents targeting these pathways. This could be very useful in cancer treatments, such as diagnosis, therapeutic targets, and basic oncology studies. For example, we can diagnose cancer early by determining sialyltransferase and sialidase levels and activities in patients. Chiang et al reported a novel sialyltransferase inhibitor AL10, which inhibits adhesion, migration, actin-polymerization, and invasion. However AL10 has no antiproliferative effect on cancer cells [39]. This phenomenon coincides with the previous pathologic view—sialyltransferases and sialidases are important in neoplasm metastasis. In the future, more sialyltransferase or sialidase inhibitors may be tested for their antimetastatic efficacies. Present sialyltransferase inhibitors (including AL10) have low specificity for distinct subclasses of sialyltransferases. Different types of sialic acid linkages elicit different types of biological responses—it will be important to design inhibitors that can selectively target specific enzymes or enzyme families (i.e. ST6GalNAcs)

Early reports showed that some antimetastatic agents greatly inhibited murine serum sias levels in mice bearing high metastatic tumors B16-F10 [40, 41]. However, the incidence of neoplasm metastases in these mice given these drugs, are reduced to very low rate. It is like the chicken-or-egg conundrum. We presently can not know which came first. But we know there is a solid relationship between them. So we need to compare all antimetastatic agents on serum sias and metastatic inhibitions to find their exact mechanism of action on neoplasm sias. This calls for strengthened research in the future.

As previously stated, there are higher levels of sialyl molecules and antigens in neoplastic or metastatic tissues. Apart from that, neoplasm tissues may carry some tumor-specific sia-analogues [42, 43]. It gives rise to an idea that monoclonal antibodies targeting these molecules should be very effective and specific in metastatic therapy. Since antibodies are important anticancer agents, especially antimetastatic agents, they are very useful in clinical therapies. A sias-targeted antibody might also be a potential therapeutic agent for metastatic treatments. This can be seen from previous references [44].

Sia—as an affinity agent to bind with highly active anticancer drugs—is an emerging drug development paradigm in cancer therapy. Since cancer cells, especially highly metastatic cells, carry some binding molecules, such as CD22, they have a high affinity to sias [45, 46]. So, we can use sias as an affinity agent to cancer cells. By chemically binding sias with anticancer drugs, we can develop more specific anticancer or antimetastatic drugs. But CD22 expression is restricted to B cells and B lymphomas. It shall be clarified that CD22 is on lymphoma cells. However, similar molecular mechanisms may occur in other cancer categories. Thus, sias is linked to anticancer drugs and these linked drugs can accumulate to higher levels in cancer cells, or metastatic cells to enhance drug activities. This way of pro-drug development is also a recent trend [47].

As some of the sialyl glyco-conjugates are unique in tumor cells, these molecules can be used as cancer vaccines. Some of them have proven to show potential as a therapeutic means, such as Theratope—a sialyl Tn derivative [48, 49]. In the future, more effective vaccines might be studied and developed.

## Discussion and Conclusion

**Fig. 1:**
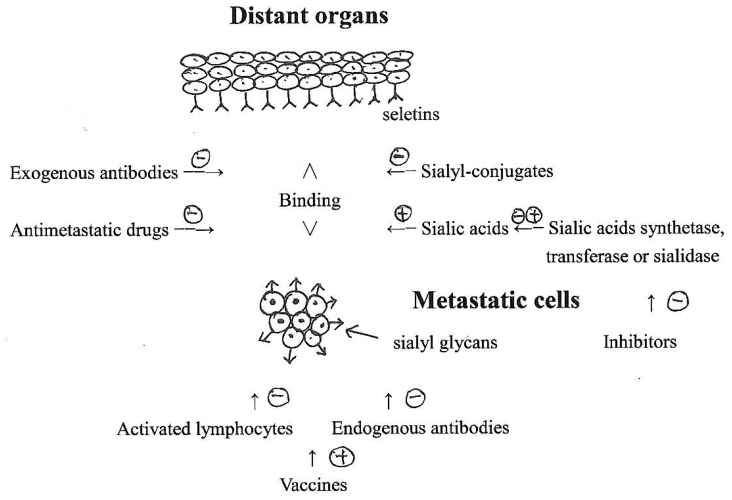
Schematic diagram of metastatic inhibitions by sialic acid-related pathways

Different pathways of antimetastatic drugs targeting neoplasm sialic acids have been concluded in table 1 and figure 1. It is the presently studied pathways that need to be strengthened. Of course new pathways are also needed. The more new targets we study, the more satisfactory results we may expect.

Since glyco-conjugates play important roles in living cells and cancer, we need to further understand them, e.g. the carbohydrate sequences that are determined by what kind of template are still not elucidated. As this “central dogma” of glycobiology is still unknown [12], some fundamental questions related to carbohydrate itself [50] are even more welcoming and decisive inour understanding of the nature of neoplasm metastasis and its inhibitions by drugs, which lead to individualized cancer chemotherapy [51]. We foresee a promising future waiting for us if we insist on this research area. Since sias are abundant sugar components, they play diversified physiological and pathological functions in a large population of living bodies, and their therapeutic drugs might lead to other biological effects. It adds much more complexity and mystery in the current perspective. It can only be known by understanding the universal theories of glycobiology.

As modern antimetastatic drug developments need new ideas and targets, the drugs targeting tumor sias seems to be a good choice. There are many ineffective antimetastatic drugs approved by authorities and some of them have been withdrawn from the market. In this critical time, we ought to consider changing our focus from the current stalemate of angiogenic therapy into some other new perspectives and insight [52–54]. Aberrantly sialylation in tumors seem to be good targets waiting for us. May we hope for the best outcomes?

## Figures and Tables

**Tab. 1:** Different pathways of antimetastatic drugs targeting neoplasm sialic acids

Compounds types	Proposed targets	Ref
Sia analogues or conjugates	Pathologic sias	22–26
DNA chelating agents	DNA template	32, 33
Sialyltransferase inhibitors	Sia adding or releasing from antigens	39
Antibodies	Pathologic antigens	46
Vaccines	Pathologic antigens	48, 49
Antimetastatic agents	Unknown mechanism	40, 41
Sia-anticancer drugs	Tumor affinity molecules	45–47
